# Unveiling the super tolerance of *Candida nivariensis* to oxidative stress: insights into the involvement of a catalase

**DOI:** 10.1128/spectrum.03169-23

**Published:** 2024-01-11

**Authors:** Yanhua Qi, Qijian Qin, Guiyan Liao, Lige Tong, Cheng Jin, Bin Wang, Wenxia Fang

**Affiliations:** 1 Institute of Biological Science and Technology, Guangxi Academy of Sciences, Nanning, Guangxi, China; 2 State Key Laboratory of Mycology, Institute of Microbiology, Chinese Academy of Sciences, Beijing, China; University of Mississippi, University, Mississippi, USA

**Keywords:** *Candida nivariensis*, H_2_O_2_-induced oxidative stress, resistance, catalase

## Abstract

Yeast cells involved in fermentation processes face various stressors that disrupt redox homeostasis and cause cellular damage, making the study of oxidative stress mechanisms crucial. In this investigation, we isolated a resilient yeast strain, *Candida nivariensis* GXAS-CN, capable of thriving in the presence of high concentrations of H_2_O_2_. Transcriptomic analysis revealed the up-regulation of multiple antioxidant genes in response to oxidative stress. Deletion of the catalase gene *Cncat* significantly impacted H_2_O_2_-induced oxidative stress. Enzymatic analysis of recombinant *Cn*Cat highlighted its highly efficient catalase activity and its essential role in mitigating H_2_O_2_. Furthermore, over-expression of *Cn*Cat in *Saccharomyces cerevisiae* improved oxidative resistance by reducing intracellular ROS accumulation. The presence of multiple stress-responsive transcription factor binding sites at the promoters of antioxidative genes indicates their regulation by different transcription factors. These findings demonstrate the potential of utilizing the remarkably tolerant *C. nivariensis* GXAS-CN or enhancing the resistance of *S. cerevisiae* to improve the efficiency and cost-effectiveness of industrial fermentation processes.

**IMPORTANCE:**

Enduring oxidative stress is a crucial trait for fermentation strains. The importance of this research is its capacity to advance industrial fermentation processes. Through an in-depth examination of the mechanisms behind the remarkable H_2_O_2_ resistance in *Candida nivariensis* GXAS-CN and the successful genetic manipulation of this strain, we open the door to harnessing the potential of the catalase *Cn*Cat for enhancing the oxidative stress resistance and performance of yeast strains. This pioneering achievement creates avenues for fine-tuning yeast strains for precise industrial applications, ultimately leading to more efficient and cost-effective biotechnological processes.

## INTRODUCTION

Yeast is a model eukaryotic microorganism that has long been used in the fermentation industry due to its manageability, safety, ease of cultivation, and reproduction (1). It is often used for ethanol fermentation and the production of specific metabolites (2, 3). During fermentation, yeast faces the challenge of adapting to environmental stress, which is considered the most challenging aspect for yeast (4). Various chemical stressors, such as ethanol, thermal, salt, and acid stress, can trigger the accumulation of reactive oxygen species (ROS) in yeast cells (5–8). This accumulation of ROS disturbs the redox homeostasis of yeast and leads to oxidative damage. Maintaining redox homeostasis is crucial for industrial yeast strains because oxidative damage during fermentation can negatively impact yeast viability and growth (9). Therefore, strategies to manage ROS levels and restore redox balance are important in industrial yeast fermentation processes. By minimizing the disruption of redox homeostasis, yeast can better withstand environmental stress and optimize its performance in fermentation.

Yeast possesses an antioxidant defense system that scavenges ROS generated by oxidative stress. This system consists of various proteins, including catalases, superoxide dismutases (Sod1 and Sod2), thioredoxins, glutathione, and peroxiredoxins (Aha1p, Tsa1p, Tsa2p, Dot5p, and Prp1p) (10–13). These proteins play critical roles in maintaining cell redox homeostasis and regulating a wide range of cellular functions. Among them, catalase (CAT) is particularly important in protecting yeast cells against oxidative damage. In *S. cerevisiae,* CAT comprises two catalase genes: peroxisomal catalase A (Cta1) and cytosolic catalase T (Ctt1) (14). Studies have shown that deficiency of Ctt1 or both Ctt1 and Cta1 impairs the adaptive response to H_2_O_2_ (15–17). Interestingly, the protein levels of peroxisomal catalase Cta1 remain unchanged, while the expression level of cytosolic catalase Ctt1 significantly increases after exposure to H_2_O_2_ stimulus (18). This suggests a different role for Cta1 and Ctt1. It is proposed that peroxisomal catalase Cta1 degrades H_2_O_2_ generated during aerobic respiration and β-oxidation, while cytosolic catalase Ctt1 reduces the accumulation of ROS generated by H_2_O_2_ stimuli. Furthermore, it has been observed that other yeast species possess catalase genes that are homologous to those found in *S. cerevisiae*. By heterologously expressing these genes, it is possible to enhance the tolerance or resistance of *S. cerevisiae* to oxidative stress. This genetic modification could potentially aid in the adaptation of *S. cerevisiae* to environmental stresses during the fermentation process (19).

While the oxidative stress system in *S. cerevisiae* has been extensively investigated, there remains a dearth of knowledge concerning non-traditional yeast species, especially *Candida nivariensis. C. nivariensis*, initially identified through DNA sequencing in 2005, has predominantly been isolated from clinical patient samples (20), with only one documented instance from fruits (21). Notably, there are no records of its industrial applications. In this study, we successfully isolated a yeast strain identified as *Candida nivariensis*, referred to as GXAS-CN, which exhibited exceptional tolerance to oxidative stress induced by H_2_O_2_. To investigate the underlying mechanism of oxidative stress tolerance in GXAS-CN, we employed RNA-Seq analysis in conjunction with knockout and phenotypic assessments. Our research unveiled the critical role of a catalase enzyme in mitigating the accumulation of ROS in GXAS-CN. Furthermore, we propose that harnessing *Cn*CAT through genetic engineering approaches could be employed to enhance oxidative tolerance in *S. cerevisiae*.

## RESULTS AND DISCUSSION

### Isolated GXAS-CN strain exhibited remarkable tolerance to oxidative stress induced by hydrogen peroxide (H_2_O_2_)

**Fig 1:**
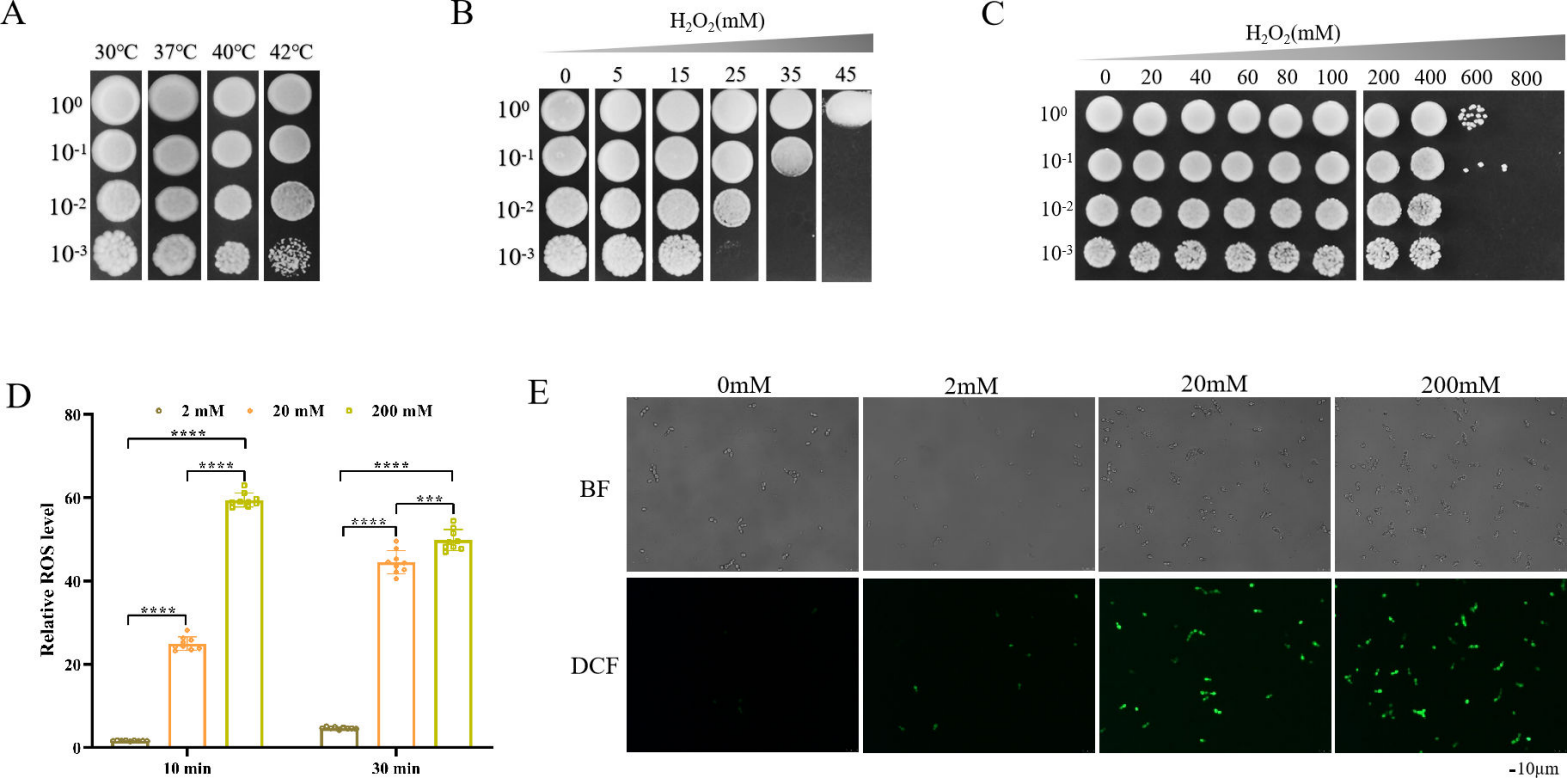
Tolerance of the GXAS-CN strain to H_2_O_2_. (**A**) Growth of GXAS-CN at different temperatures. Mid-log phase yeast cells were inoculated on YPD plates and incubated at various temperatures for 2  days. (**B**) Remarkable tolerance of GXAS-CN to H_2_O_2_-induced oxidative stress. Mid-log phase cells were inoculated on YPD plates containing different concentrations of H_2_O_2_ and incubated at 37°C for 2  days. (**C**) Cell survival of GXAS-CN following H_2_O_2_ exposure. Mid-log phase cells were exposed to H_2_O_2_ for 3 hours with shaking, then serially diluted and incubated on YPD plates at 30°C for 2  days. (**D**) Measurement of intracellular ROS levels in the GXAS-CN strain after 10 min or 30 min of H_2_O_2_ exposure. (***P < 0.001; ****P < 0.0001.) (**E**) DCF Fluorescence observation of GXAS-CN cells after 30 min of H_2_O_2_ shock. The scale bar is 10 µm.

The ability to withstand oxidative stress is critical for yeast cells used in industrial applications. Previous studies have shown that yeast species generally show low tolerance to H_2_O_2_-induced oxidative stress, resulting in inhibited growth and reduced survival rates (22, 23). In this study, we isolated a strain displaying exceptional tolerance to H_2_O_2_. Following DNA extraction and sequencing of the strain's ITS sequence and the conservative D1/D2 region of 26S ribosomal DNA (24), a phylogenetic tree was constructed using MEGA X software. The analysis revealed that the strain belonged to the *Candida nivariensis* species and was referred to as GXAS-CN (Fig. S1). While it is important to note that *C. nivariensis* has the potential to act as an opportunistic pathogen, it is crucial to emphasize that the associated risks when working with such potential pathogens can be effectively mitigated through the rigorous implementation of biosafety principles and best practices. Further characterization of GXAS-CN revealed its ability to grow across a wide temperature range, including 30°C , 37°C , 40°C , and 42°C, with particularly robust growth observed at 42°C (Fig. 1A). Moreover, GXAS-CN exhibited normal viability even under high concentrations of H_2_O_2_ (Fig. 1B) and maintained higher cell viability when treated with 400 mM H_2_O_2_ for 3 hours (Fig. 1C). In comparison to other yeasts, including *S. cerevisiae*, *C. shehatae*, *C. succiphila*, and *Pichia guillermondii*, which exhibit inhibition at a 5 mM H_2_O_2_ concentration (23), our findings highlight the exceptional oxidative stress tolerance of the GXAS-CN strain.

Various stresses, including H_2_O_2_-induced oxidative stress and high temperature, are known to induce the accumulation of reactive oxygen species (ROS) in cells. ROS accumulation has been shown to have detrimental effects on yeast viability, growth, and fermentation profiles (25). To investigate the relationship between ROS accumulation and oxidative stress in GXAS-CN, we measured the intracellular ROS levels at 10 min and 30 min following exposure to H_2_O_2_ (Fig. 1D). Our results demonstrated that treatment with H_2_O_2_ led to a substantial accumulation of DCF-sensitive intracellular ROS, both at concentrations of 20 mM and 200 mM H_2_O_2_, after 10 min or 30 min of treatment. These findings were further supported by fluorescence observation taken after 30 min of H_2_O_2_ exposure (Fig. 1E). These results indicate that GXAS-CN experiences a significant increase in intracellular ROS levels and signaling upon H_2_O_2_ induction; yet, the strain exhibits a remarkable ability to survive under such conditions.

Furthermore, GXAS-CN exhibited remarkable tolerance to various by-products generated during lignocellulosic material pretreatment, saccharification steps, and ethanol fermentation. These by-products included 10% ethanol, 10 mM vanillin, 80 mM acetic acid, 35 mM formic acid, and 25 mM furfural (Fig. S2). The strain exhibited robust viability and growth even in the presence of these inhibitory compounds, underscoring its potential for efficient fermentation under challenging conditions.

### Transcriptome analysis revealed up-regulated expression of several antioxidases responding to oxidative stress in GXAS-CN

**Fig 2:**
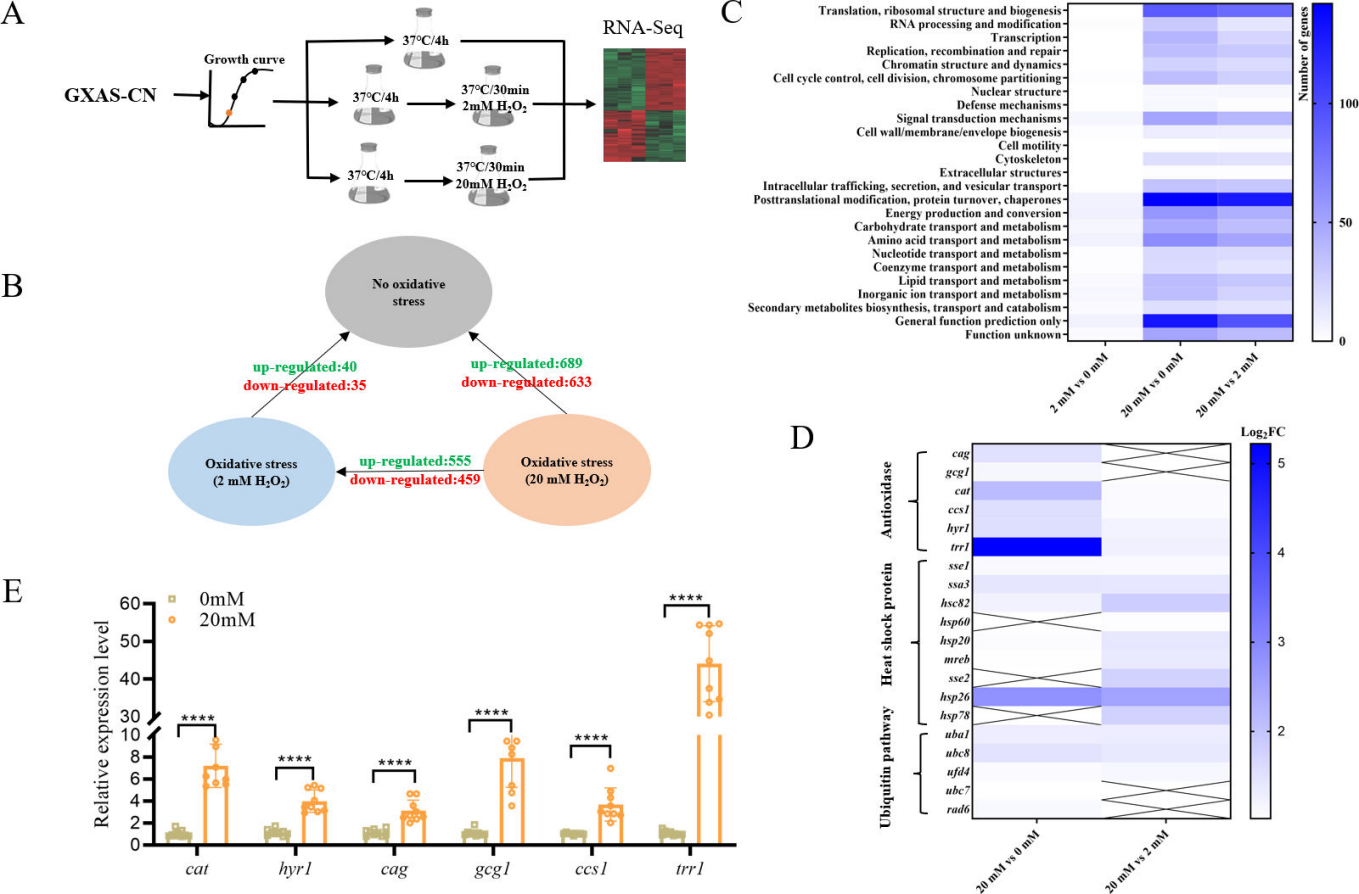
Transcriptome analysis of GXAS-CN under different concentrations of H_2_O_2_ treatment. (**A**) Schematic representation of the RNA-Seq process under H_2_O_2_-stressed and unstressed conditions. (**B**) The number of differentials expressed genes under H_2_O_2_-stressed and unstressed conditions. (**C**) COG analysis of regulated metabolic genes under H_2_O_2_-stressed and unstressed conditions. (**D**) Transcriptional profile of related genes, including those associated with the antioxidant defense system, heat shock proteins, and the ubiquitin pathway. (**E**) Expression of oxidative stress-related genes (*cag*, *gcg1*, *cat*, *ccs1*, *hyr1*, *trr1*) in GXAS-CN. The gene expression levels under 20 mM H_2_O_2_-stressed and unstressed conditions were measured by qRT-PCR (****P < 0.0001).

Since the GXAS-CN strain exhibited remarkable tolerance to H_2_O_2_-induced oxidative stress, we aimed to gain a deeper understanding of the molecular mechanism underlying its resistance to oxidative stress. To achieve this, we sequenced the entire genome of GXAS-CN and conducted RNA-seq analysis to examine the global gene expression profiles. According to the illustrated treatment protocol (Fig. 2A), we analyzed the expression levels of 5,057 annotated functional genes across the entire genome. Differential gene expression was performed using a false discovery rate (FDR) threshold of FDR  ≤0.01 and a fold change cutoff of ≥2. The comparison of transcriptome data between untreated GXAS-CN and those treated with 2 mM H_2_O_2_ did not reveal significant differences in gene expression. However, when exposed to 20 mM H_2_O_2_ treatment, a notable transcriptional response was observed. Specifically, 689 genes were up-regulated, while 633 genes were down-regulated (Fig. 2B). These results highlight that GXAS-CN exhibits a specific transcriptional response to high levels of H_2_O_2_-induced oxidative stress.

Functional pathways within GXAS-CN were explored through COG function classification, revealing a diverse array of pathways influenced by high concentrations of H_2_O_2_, with over 50 differentially expressed genes (DEGs) associated with these pathways. These pathways included translation, ribosomal structure and biogenesis, posttranslational modification, protein turnover, chaperones, carbohydrate transport and metabolism, and amino acid transport and metabolism. Furthermore, in cells treated with low concentrations of H_2_O_2_, specific pathways such as energy production and conversion, carbohydrate transport and metabolism, and amino acid transport and metabolism were affected (Fig. 2C; Table S2). Upon analyzing the regulated genes, notable up-regulation was observed in peroxisomal proteins, heat shock proteins, and proteins involved in the ubiquitin pathway (Fig. 2D; Table S2). Specifically, the peroxisomal catalase gene *cat* and the pyridine nucleotide-disulfide oxidoreductase gene *trr1*, the heat shock protein gene, and genes associated with the ubiquitin protease pathway exhibited significant upregulation. This upregulation plays a critical role in reducing intracellular ROS levels, repairing misfolded proteins, and degrading damaged or misfolded proteins. To validate the RNA-seq data, we selected six upregulated antioxidant genes and assessed their expression changes using qRT-PCR (Fig. 2E). The results not only confirmed the reliability of the differential gene expression analyses based on RNA-seq and underscored the significant role of those antioxidant genes, which contributes to the oxidative stress tolerance of GXAS-CN.

### Heterologous overexpression of the *Cncat* gene in *S. cerevisiae* resulted in increased resistance to H_2_O_2_-induced oxidative stress

**Fig 3:**
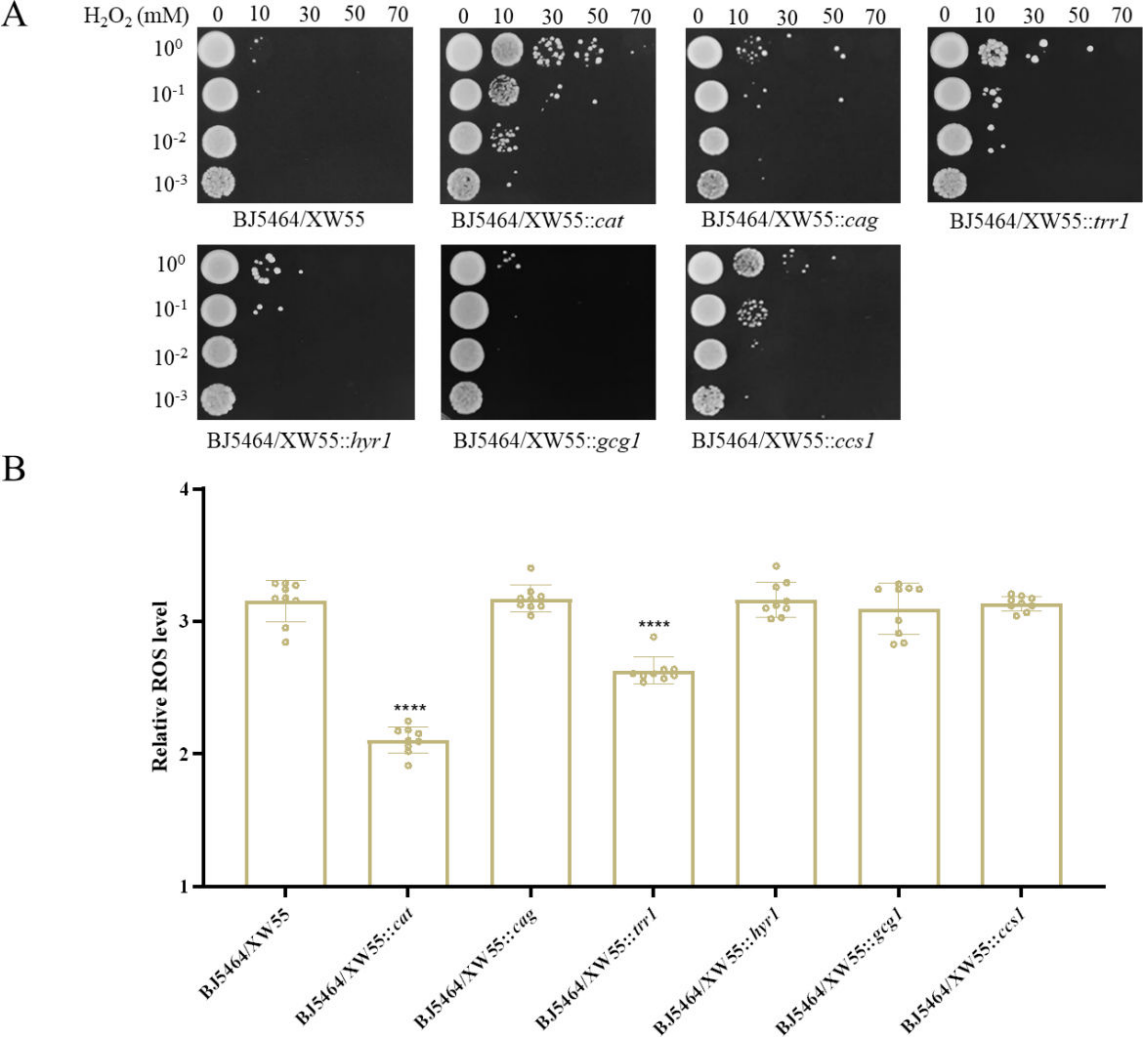
Assessment of cell survival of *S. cerevisiae* strains with heterologously overexpressed antioxidant genes following H_2_O_2_ exposure. (**A**) Mid-log phase yeast cells were subjected to a 3 hour treatment with shaking in the presence of H_2_O_2_. The treated cells were serially diluted and inoculated on YPD plates. The plates were incubated at 30°C for 2  days. (**B**) The intracellular ROS levels were measured in *S. cerevisiae* strains that overexpressed antioxidant genes after a 30 min shock with 20 mM H_2_O_2_ (****P < 0.0001).

The natural traits of *S. cerevisiae* for adapting to harsh fermentation conditions are often inadequate for industrial applications. Previous studies have demonstrated that heterologous expression of stress-related genes, such as *Dh*Cta1 or *Dh*Ctt1, improves tolerance to H_2_O_2_-induced oxidative stress (26), while expression of *Km*Hsf1 or *Km*Msn2 enhances cell growth and ethanol fermentation at high temperatures (27). Therefore, introducing exogenous stress-related genes has been explored as an effective approach to enhance stress tolerance. Based on our transcriptome data, we propose that heterologous overexpression of six regulated antioxidant genes from GXAS-CN can enhance *S. cerevisiae*'s resistance to H_2_O_2_-induced oxidative stress. To test this hypothesis, we introduced plasmid XW55, which carries the coding sequences of the six antioxidant genes, into *S. cerevisiae* and verified through growth on selective plates and PCR confirmation. As shown in Fig. 3A, yeast cells expressing *cat*, *trr1*, and *ccs1* exhibited significantly improved cell viability when exposed to H_2_O_2_, in comparison to the control strain. Furthermore, when measuring intracellular ROS accumulation following H_2_O_2_ exposure, cells expressing *cat* and *trr1* effectively reduced ROS levels (Fig. 3B). This reduction signifies an improved tolerance to oxidative stress, primarily achieved through ROS reduction, likely through mechanisms involving the breakdown of H_2_O_2_ or the provision of reducing power to support thioredoxins and peroxiredoxins (18, 28).

Considering the positive effect of single antioxidant gene heterologous expression on H_2_O_2_ tolerance, we hypothesized that the simultaneous expression of two antioxidant genes might exert a more robust effect. To test this hypothesis, we constructed strains expressing the *cat* gene along with other antioxidant genes and assessed their ability to improve tolerance to H_2_O_2_-induced oxidative stress using cell viability assays. The results revealed that *Cn*Cat could potentially exhibit a synergistic effect with other antioxidant proteins in *S. cerevisiae*. These engineered strains exhibited significantly higher survival rates after 3 hours of 70 mM H_2_O_2_ treatment compared to the control strain (Fig. S4A). In addition, intracellular ROS accumulation demonstrated lower ROS levels in these strains under oxidative stress conditions compared to the control strain (Fig. S4B). These findings provide compelling evidence that the heterologous overexpression of the *cat* gene, either individually or in combination with other genes, in *S. cerevisiae* can indeed bolster resistance to H_2_O_2_-induced oxidative stress.

### The catalase *Cn*Cat is integral for resistance to oxidative stress in GXAS-CN

**Fig 4:**
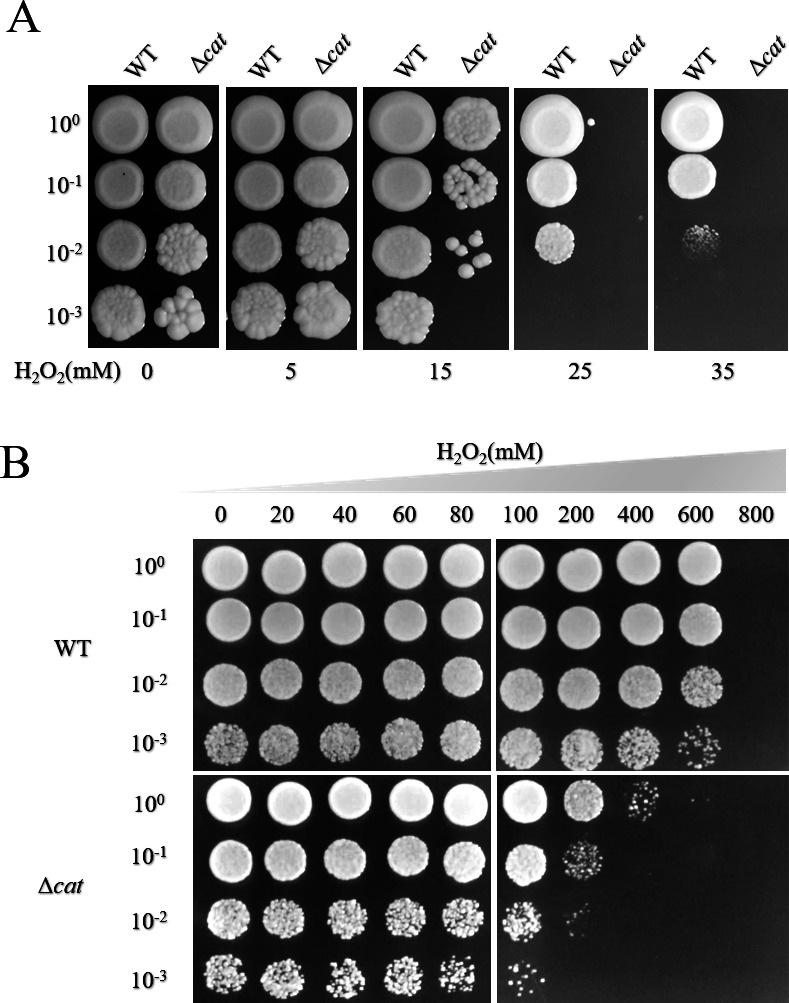
Impact of *Cncat* deletion mutants on oxidative stress resistance in GXAS-CN. (**A**) Sensitivity of *Cncat* deletion mutants to H_2_O_2_. Mid-log phase cells were inoculated on YPD plates supplemented with various concentrations of H_2_O_2_. The plates were then incubated at 37°C for 2  days. (**B**) The assessment cell survival in *Cncat* deletion mutants exposed to H_2_O_2_. Mid-log phase cells were exposed to H_2_O_2_ for 3 hours with shaking, then serially diluted and inoculated on YPD plates. The plates were incubated at 30°C for 2  days.

The heterologous overexpression of the *Cncat* gene significantly inhibits H_2_O_2_-induced ROS accumulation in *S. cerevisiae*. However, the specific role of *Cn*Cat in GXAS-CN remained elusive. To investigate its function in oxidative stress, we constructed an in-frame deletion mutant removing the *Cncat* gene, which was confirmed by PCR analysis (Fig. S3). This marked the first successful genetic manipulation in *C. nivariensis,* and we named the resulting mutant Δ*cat*. Subsequent growth assays on YPD plates supplemented with various concentrations of H_2_O_2_ revealed that deletion mutant Δ*cat* exhibited growth impairments, particularly evident under 15 mM and 25 mM H_2_O_2_, in comparison to the control strain (Fig. 4A). Furthermore, cell viability assays demonstrated that the deletion mutants displayed reduced tolerance to H_2_O_2_ compared to the wild type (Fig. 4B). Interestingly, unlike *Sc*Cta1 in *S. cerevisiae*, which primarily clears cytosolic ROS, and *Sc*Ctt1, which deals with oxidative stress from external sources (29–31), the catalase *Cn*Cat in GXAS-CN plays a dominant role in combating H_2_O_2_-induced oxidative stress, as supported by RNA-seq data and the deletion mutant experiments.

Catalases are predominantly localized within peroxisomes and typically feature a peroxisomal targeting signal (PTS) that directs their specific peroxisome localization. González et al. reported that *Sc*Cta1 and *Dh*Cta1, catalases from *S. cerevisiae* and *D. hansenii* (19), respectively, have their PTS targeting signals located at the extreme C-terminus of their protein sequences. However, Petrova et al. and Rymer et al. have noted that other peroxisomal proteins lack the typical PTS1 (SSNSKF) or PTS2 (SKF) signals (32, 33). In the case of *Cn*Cat, no PTS targeting signal was identified in the C-terminus of its protein sequence (Fig. S5). However, we did find a target signal sequence PTS2 (SKF) within the N-terminal region of the *Cn*Cat protein, suggesting that *Cn*Cat may rely on unknown PTS signals or alternative mechanisms, resulting in its significant impact on both the cytoplasm and peroxisomes. Further studies are needed to elucidate the precise mechanisms and signals involved in the peroxisomal targeting of *Cn*Cat in GXAS-CN.

### The catalase *Cn*Cat from GXAS-CN exhibits high activity

**Fig 5:**
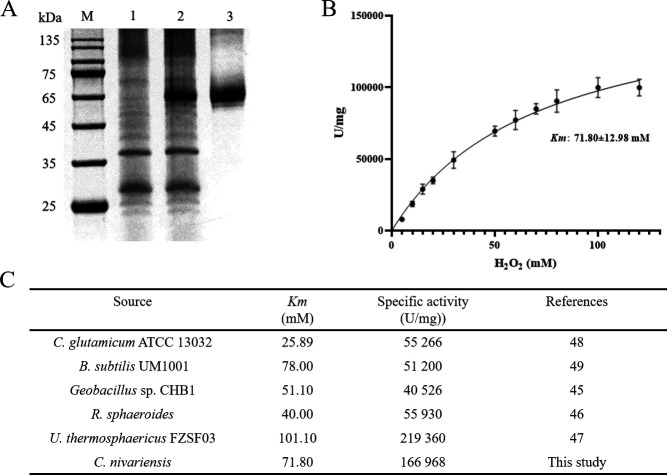
The enzyme properties of *Cn*Cat from GXAS-CN. (**A**) Expression and purification of His-*Cn*Cat. Lane 1, the lysate without His-*Cn*Cat expression; lane 2, the lysate with His-*Cn*Cat expression; lane 3, the purified His-*Cn*Cat protein. A molecular weight marker (**M**) was included for reference. (**B**) The kinetic curve of the recombinant catalase His-*Cn*Cat. Enzyme activity was measured under optimal pH and temperature conditions. (**C**) The catalase activity of *Cn*Cat was compared to catalases from various sources (34–38).

To investigate the role of catalase *Cn*Cat in ROS elimination and cellular protection, we constructed a recombinant *E. coli* strain overexpressing the His-*Cn*Cat fusion protein and successfully expressed and purified the protein. SDS-PAGE analysis of the purified His-*Cn*Cat protein under denaturing conditions revealed an estimated molecular weight of 65 kDa (Fig. 5A). To measure the catalase activity of *Cn*Cat, we employed the catalase-H_2_O_2_ system. The results, shown in Fig. 5B and Fig. S6, demonstrate that His-*Cn*Cat exhibits a favorable substrate affinity, following the classical Michaelis-Menten equation under optimal pH and temperature conditions. Its specific activity surpasses that of catalases from various sources, reaching levels as high as 166,968 U/mg, second only to the catalase from the thermophilic bacterium *Ureibacillus thermosphaericus* (Fig. 5C). These findings highlight the remarkable efficiency of catalase *Cn*Cat in decomposing H_2_O_2_ and reducing intracellular ROS accumulation. The high specific activity of *Cn*Cat underscores its potential as a highly effective enzyme for ROS detoxification and oxidative stress management.

Furthermore, we performed homology modeling of *Cn*Cat and observed that it adopts a classical homotetrameric structure, similar to peroxisomal catalases found in *P. pastoris* and *Kluyveromyces lactis* (39). This homotetramer is composed of four distinct structural elements: the N-terminal arm, the central domain, the wrapping loop, and the C-terminal domain. The active sites, crucial for catalytic activity, are located within the homotetramer's pocket (Fig. S7A). The quality of the structural model was assessed using the Ramachandran plot, which confirmed the model’s credibility (Fig. S7B).

### The promoters of antioxidant genes in GXAS-CN exhibit multiple transcription factor (TF)-binding sites involved in stress response

**Fig 6:**
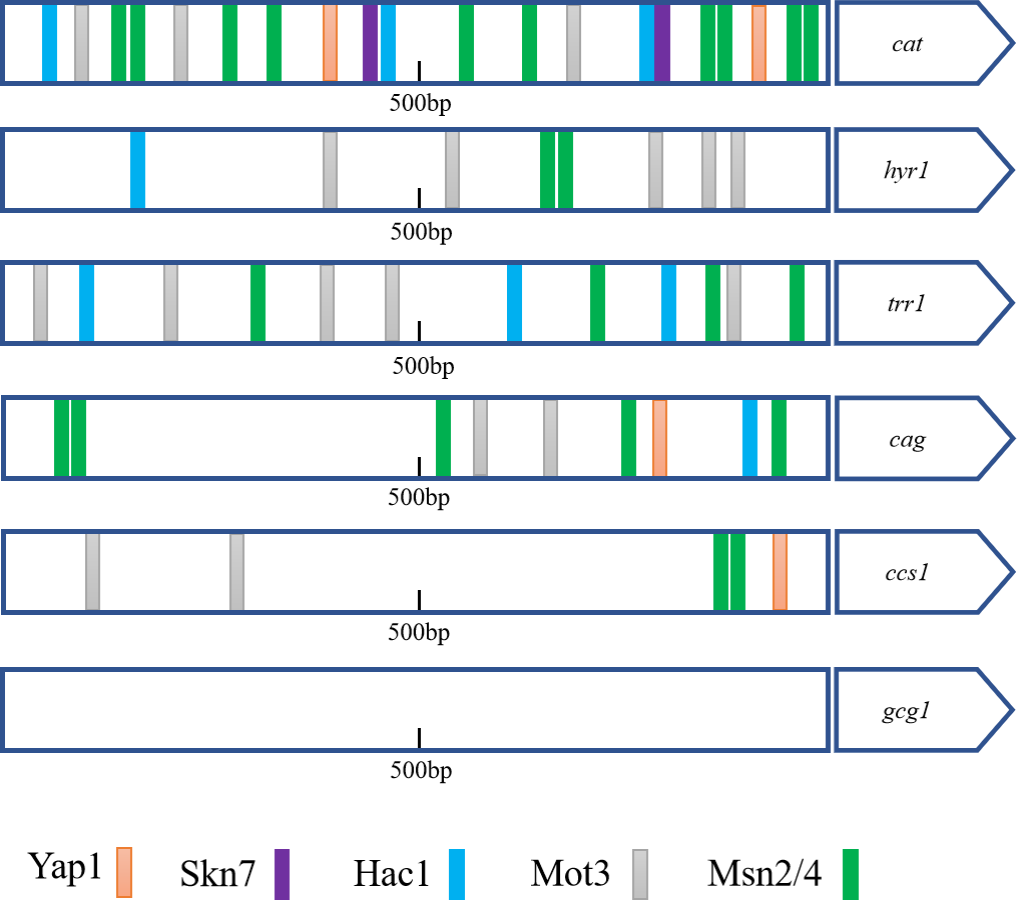
Prediction of stress transcription factor binding sites in the promoters of genes encoding antioxidant enzymes. TF binding sites are indicated as color-coded boxes.

Catalase *Cn*Cat plays a crucial role in oxidative stress resistance in GXAS-CN*,* attributed not only to its high enzymatic activity but also to its high gene expression level. However, how the regulatory mechanisms governing the expression of *Cn*Cat and other antioxidant genes in GXAS-CN remain unknown. To investigate whether stress transcription factors (TFs) are involved in their regulation, we analyzed the 1,000 bp promoter sequences of antioxidant genes, using *S. cerevisiae* transcription factor binding sites as a reference (Fig. 6). Examples include the TTACTAA sequence recognized by Yap1 (26), the GGC(C/T) GGC sequence recognized by Skn7 (40), the CCAGC sequence recognized by Hac1 (41), the (C/A/T)AGG(T/C)A sequence recognized by Mot3 (42), and the CCCCN sequence recognized by Msn2/4 (26). Yap1 is activated under oxidative stress, localizes to the nucleus, and promotes the transcription of antioxidant genes (43). Skn7 is specifically involved in responses to osmotic and oxidative stresses, often in conjunction with Yap1 (13, 44). Hac1 regulates the transcription of unfolded protein response (UPR) target genes and is involved in mediating the derepression of certain genes under metal-induced oxidative stress in coordination with Yap1 (45). Mot3 and Rox1 regulate the expression of ergosterol biosynthesis genes under high osmolarity or salt stress (46). Msn2/4 controls the expression of 27 proteins, enhancing H_2_O_2_ tolerance (47).

Our analysis revealed that the promoters of antioxidant genes contain several stress response-associated transcription factor (TF)-binding sites. Notably, the promoter of the *Cncat* gene exhibits a great number of TF-binding sites compared to other antioxidant genes, suggesting that the high expression of the *Cn*Cat gene may be regulated by different transcription factors under various stress conditions. In addition, apart from *gcg1*, the promoters of other antioxidant genes (*hyr1*, *trr1*, *cag1,* and *ccs2*) also contain multiple binding sites of these TFs, including Yap1, Msn2/4, and Skn7. These TFs have been shown to enhance the expression of antioxidant genes (such as *cat*, *trr1*) in response to oxidative stress in other organisms like *C. glabrata* (48). These findings highlight the significant role of stress transcription factors in GXAS-CN in regulating the expression of antioxidant genes.

### Conclusion

In conclusion, our study focused on the *Candida nivariensis* GXAS-CN yeast strain and its response to H_2_O_2_-induced oxidative stress. We discovered that the catalase *Cn*Cat played a crucial role in enhancing the strain's tolerance to oxidative stress. Heterologous overexpression of *Cn*Cat in *S. cerevisiae* resulted in improved cell viability under H_2_O_2_ shock, demonstrating its potential as a key factor in oxidative stress response and its applicability in biotechnological applications within the fermentation industry. Furthermore, our investigation shed light on the regulatory mechanisms involved in antioxidant gene expression, with the promoters of these genes containing multiple stress-responsive transcription factor binding sites. Notably, the *Cn*Cat gene promoter exhibited a higher number of such binding sites compared to other antioxidant genes, suggesting its potential regulation by different transcription factors under various stress conditions. This knowledge can be leveraged to optimize biotechnological processes in the fermentation industry, enabling the more efficient production of valuable compounds.

## MATERIALS AND METHODS

### Strain isolation

A total of 216 strains were isolated from different samples collected in Guangxi Province and subjected to screening for their growth capability on YPD plates supplemented with 10 mM H_2_O_2_. Remarkably, only a single strain obtained from a spoiled fruit displayed vigorous growth. The specific strain was designated as GXAS-CN and further identified as *Candida nivariensis* through ITS and D1/D2 region amplification.

### Growth characterization of GXAS-CN under various stress conditions

The growth characteristics of GXAS-CN were examined under various conditions. Initially, yeast cells were cultured in a YPD medium until they reached an optical density at 600 nm (OD_600_) of 1. Subsequently, 3 µL of aliquots of 10^0^, 10^−1^, 10^−2^, and 10^−3^ dilutions was applied onto YPD plates. These plates were then incubated at different temperatures (30°C, 37°C, 40°C, and 42°C) for a duration of 2 days, and the growth response was evaluated.

To investigate the impact of oxidative stress on the growth of GXAS-CN, cells grown to an OD_600_ of 1 in a YPD medium were subjected to 3 µL of aliquots of 10^0^, 10^−1^, 10^−2^, and 10^−3^ dilutions, which were inoculated onto YPD plates supplemented with various concentrations of H_2_O_2_. The plates were then incubated at a constant temperature of 37°C for 2 days.

To assess cell viability, yeast cells grown to an OD_600_ of 1 in a YPD medium were subjected to various concentrations of H_2_O_2_ (ranging from 0 to 800 mM) for 3 hours at 30°C with shaking. After the H_2_O_2_ treatment, the cells were washed twice with water and then inoculated onto YPD plates following serial dilutions (10^0^, 10^−1^, 10^−2^, and 10^−3^). The plates were subsequently incubated at 30°C for 2 days to evaluate cell viability under these conditions.

### Detection of ROS production and accumulation

To investigate ROS production and accumulation under different oxidative stress conditions, yeast cells were cultured in a YPD medium until they reached an OD_600_ of 1. The cells were then harvested, washed with PBS buffer, and incubated with dichlorodihydrofluorescein diacetate (DCFH-DA) at 30°C for 30 min. After centrifugation, washing, and dilution with PBS buffer, the intracellular ROS levels were assessed by measuring fluorescence intensity at an excitation wavelength of 488 nm and an emission wavelength of 525 nm. This measurement was conducted following the addition of H_2_O_2_ at concentrations ranging from 0 to 200 mM for either 10 min or 30 min. Furthermore, fluorescence microscopy was utilized to observe the green fluorescence emitted by DCF, which indicates the presence of ROS. This observation was made after incubating the yeast cells with H_2_O_2_ concentrations ranging from 0 to 200 mM for 30 min.

### Global transcriptomic analysis of GXAS-CN under oxidative stress conditions

For the global transcriptional analysis, the GXAS-CN cells were cultured in a fresh medium until they reached the mid-exponential phase (OD_600_≈0.8) at 37°C. Subsequently, either 2 mM or 20 mM H_2_O_2_ was added to the culture, and the cells were further incubated for an additional 30 min. The cells were then collected through suction filtration and washed with RNA-free water. The collected cells were sent to the BioMarker company (Beijing, China) for sample extraction and sequencing. Total RNA was isolated from the cells, and its quantity and quality were assessed using a Nanodrop spectrophotometer ND-8000 and Agilent 2100 bioanalyzer, respectively. RNA sequencing was performed on an Illumina NovaSeq6000 platform.

### Quantitative real-time reverse transcription PCR

To validate the expression patterns of specific genes, quantitative real-time reverse transcription PCR (qRT-PCR) was performed. The cDNA was synthesized from the total RNA of the collected samples using the HiScript III 1st Strand cDNA Synthesis kit (Vazyme) according to the manufacturer's instructions. For qRT-PCR, various primer sets were used, and the synthesized cDNA was used as the template. The primer sequences for qRT-PCR are listed in Table S1, with *act1* of *C. nivariensis* serving as the internal control gene. The relative expression level of the target gene was calculated using the 2^-ΔΔCt^ method.

### Heterologous expression of GXAS-CN antioxidant genes in *S. cerevisiae*

The open reading frames (ORFs) of the antioxidant gene were amplified by PCR using the respective templates (Table S1) and then cloned into the XW55 vector to generate recombinant plasmids. Subsequently, these recombinant plasmids were introduced into *S. cerevisiae* BJ5464 through chemical transformation, leading to the generation of recombinant strains referred to as BJ5464/XW55::*genes*. For instance, a strain expressing the *cat* gene heterologously was designated as BJ5464/XW55::*cat*.

### Construction of catalase deletion mutant in GXAS-CN

The catalase deletion mutant in GXAS-CN was constructed by homologous recombination. To generate the deletion construct, two genomic fragments were amplified from GXAS-CN genomic DNA. The first fragment was a 963 bp segment upstream of the *cat* gene, and the second fragment was a 962 bp segment downstream of the *cat* gene. A 1,120 bp *natMX* gene, serving as the selection marker, was amplified from the plasmid pFA6a-Flag-NatMX. Subsequently, these three fragments were seamlessly connected to form the upstream-*natMX*-downstream fragment. The resulting fragment was then introduced into GXAS-CN cells through the chemical transformation process. The transformed cells were screened on YPD plates containing 200 µg/mL NatMX. The resulting *cat* deletion mutant was confirmed through PCR analysis and designated as Δ*cat* (Fig. S3).

### Recombinant expression and purification of *Cn*Cat

To overexpress *Cn*Cat in *E. coli*, the coding sequence of *Cn*Cat protein was amplified by PCR using gene‐specific primers and cloned into the pET28a vector. The resulting plasmid was subsequently introduced into BL21(DE3)plys cells, which allowed for the production of the His-tagged fusion protein, His-*Cn*Cat. After induction of the fusion protein using 0.5 mM IPTG at 37 ℃ for 6 hours, the cells were collected and subjected to ultrasonication in a binding buffer (50 mM NaH_2_PO_4_, 150 mM NaCl, 0.5 mM TCEP, pH 7.5). The resulting lysate was then centrifuged, and the supernatant was passed through a TALON His-Tag (Takara) column. To remove nonspecifically bound proteins, the column was washed with 10 volumes of wash buffer (20 mM imidazole in binding buffer). Subsequently, the purified recombinant protein, His-*Cn*Cat, was eluted from the column using an elution buffer (150 mM imidazole in binding buffer). To remove any remaining high concentrations of imidazole, the purified His-*Cn*Cat was subjected to ultrafiltration using 50  mM PBS buffer (pH 7.0). Finally, the concentration of the purified recombinant protein His-*Cn*Cat was determined using the Bradford method.

### Catalase activity assay

The catalase activity assay was performed according to the described method (49). In this assay, 10 µL of diluted His-*Cn*Cat protein was added to a mixture containing 190 µL of 80 mM H_2_O_2_ in 50  mM PBS buffer (pH 8.0). The decomposition of H_2_O_2_ was then monitored at 240 nm. Catalase activity is defined as one unit when it catalyzes the decomposition of 1 µM H_2_O_2_ per minute, and results are presented as the average catalase activity ± standard deviation (SD) obtained from three independent cultures (*n* = 3) (26).

### Characterization of His-*Cn*Cat

For the assessment of optimum pH, the activities of the purified recombinant protein His-*Cn*Cat were measured in buffers with different pH values ranging from 3 - 11. Specifically, pH 3-5 used a 50  mM sodium citrate buffer, pH 6-8 used a 50 mM sodium phosphate buffer, pH 9 used a 50 mM Tris-HCl buffer, and pH 10-11 used a 50 mM NaHCO_3_-NaOH buffer. To determine the enzyme's optimum temperature, the activities of the purified recombinant protein His-*Cn*Cat were measured at temperatures ranging from 20 to 50°C.

### Structure modeling of *Cn*Cat

The structural model of *Cn*Cat was generated by utilizing the catalase from *Kluyveromyces lactis* (PDB: 6RJR) as a template, which exhibited an 81% sequence identity and covered 97% of the template structure. This model was constructed with the assistance of SWISS-MODEL (Fig. S7A). Furthermore, the quality of the structural model was assessed using the Ramachandran plot, which confirmed the model’s credibility (Fig. S7B).

## Data Availability

The resulting FASTQ files associated with this project have been deposited in the NCBI database under accession number PRJNA944408.

## SUPPLEMENTAL MATERIAL

Supplemental material spectrum.03169-23-s0001.docx Fig. S1 to S7 and Tables S1 to S3.

ASM does not own the copyrights to Supplemental Material that may be linked to, or accessed through, an article. The authors have granted ASM a non-exclusive, world-wide license to publish the Supplemental Material files. Please contact the corresponding author directly for reuse.
