# Supplementary material for: Unveiling the super tolerance of Candida nivariensis to oxidative stress: insights into the involvement of a catalase
Source: Microbiol Spectr. 2024 Jan 11;12(2):e03169-23. doi: 10.1128/spectrum.03169-23 (PMC10846165; doi:10.1128/spectrum.03169-23)
Supplement: Supplemental material — Fig. S1 to S7 and Tables S1 to S3. [file spectrum.03169-23-s0001.docx]

A

**B**

**Fig S1 Phylogenetic tree showing that the isolated strain belongs to *Candida nivariensis, as* inferred from ITS sequence (A) and D1/D2 region (B).** The tree was constructed using the neighbor-joining method where the numbers on the tree represent points of divergence in organisms that produce new gene copies or share a common ancestor, and the scale bar represents 0.05 substitutions per nucleotide.


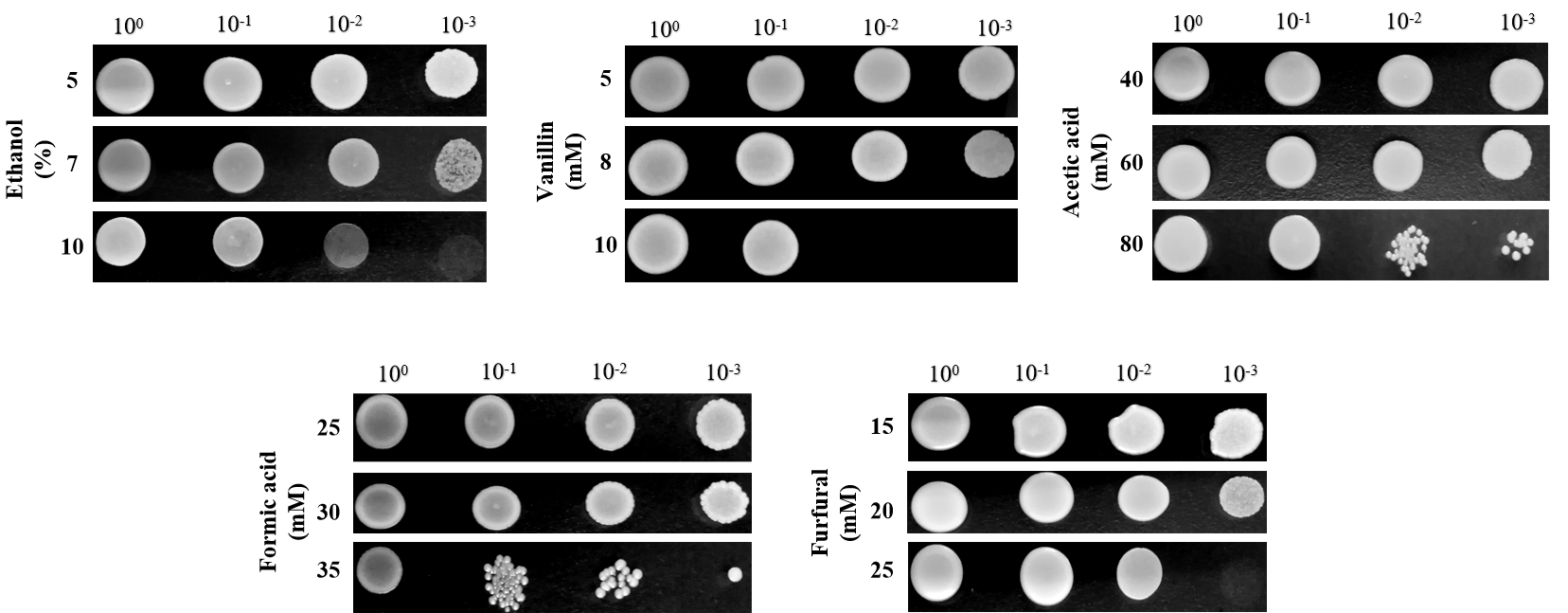


**Fig. S2 Effect of other stresses on the growth of GXAS-CN strain.** Mid-log phase yeast cells were inoculated onto YPD plates containing stress factors with different concentrations. Plates were incubated at 37 °C for 2 days.


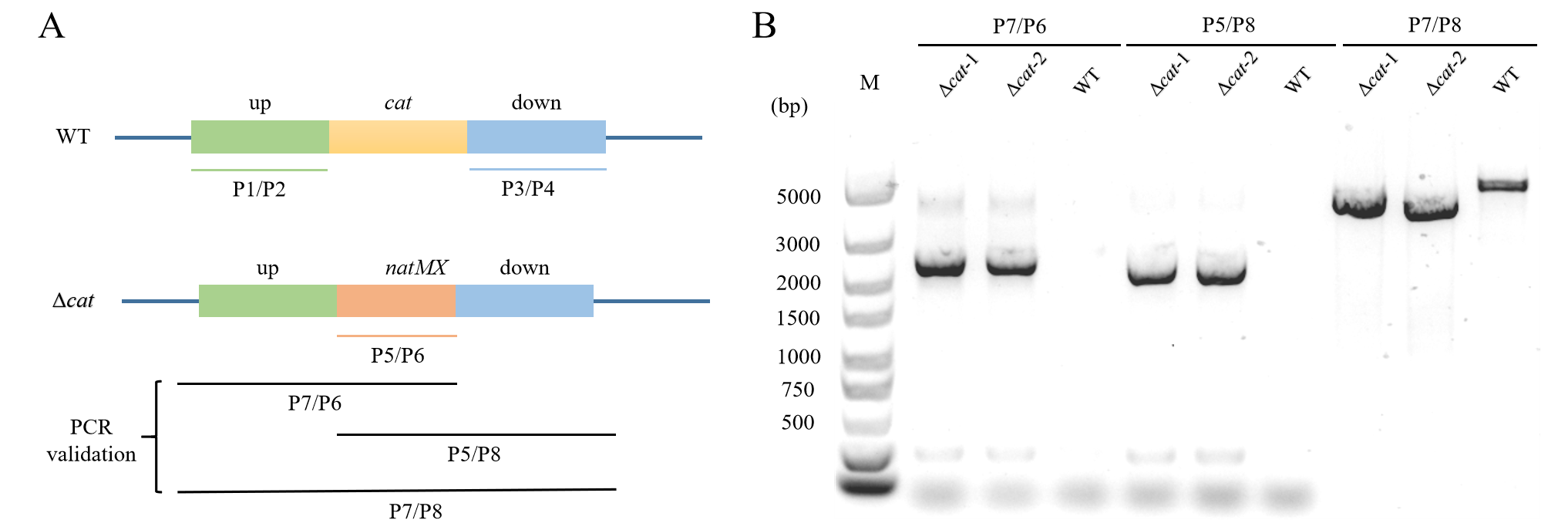


**Fig S3 PCR analysis of Δ*cat* mutant.**

A Diagram illustrating strategies of constructing the Δ*cat* mutant.

B Confirmation of the mutant strains by PCR using primers P7/P6, P5/P8 and P7/P8 as shown in A.


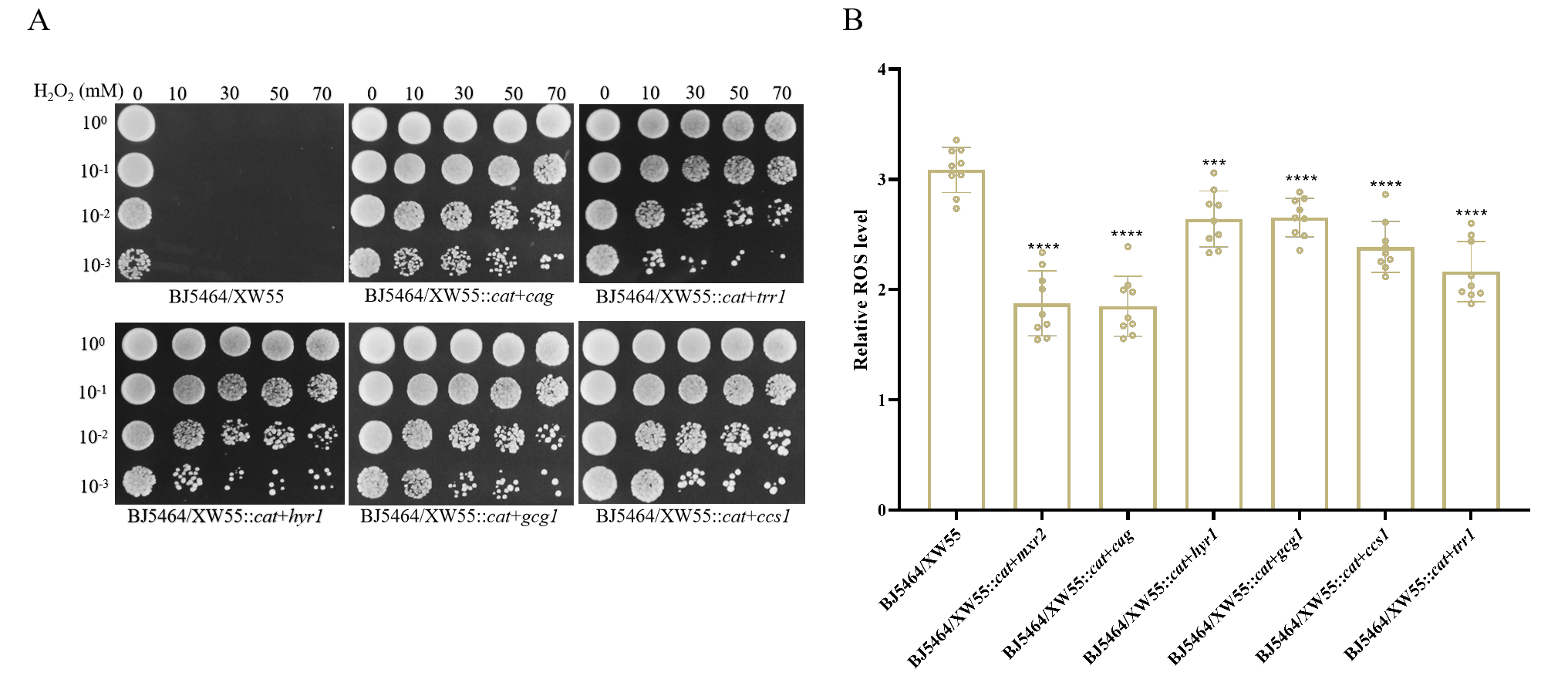


**Fig S4 Assessment of cell survival of *S. cerevisiae* strains with heterologously overexpressed antioxidant genes following H_2_O_2_ exposure.**

A Mid-log phase yeast cells were exposed to H_2_O_2_ for 3 h with shaking and inoculated onto YPD plates serially. The plates were incubated at 37 °C for 2 days.

B Intracellular ROS levels were measured in *S. cerevisiae* strains overexpressing antioxidant genes after a 30 min shock with H_2_O_2_. (***, *p*<0.001; ****, *p*<0.0001.)


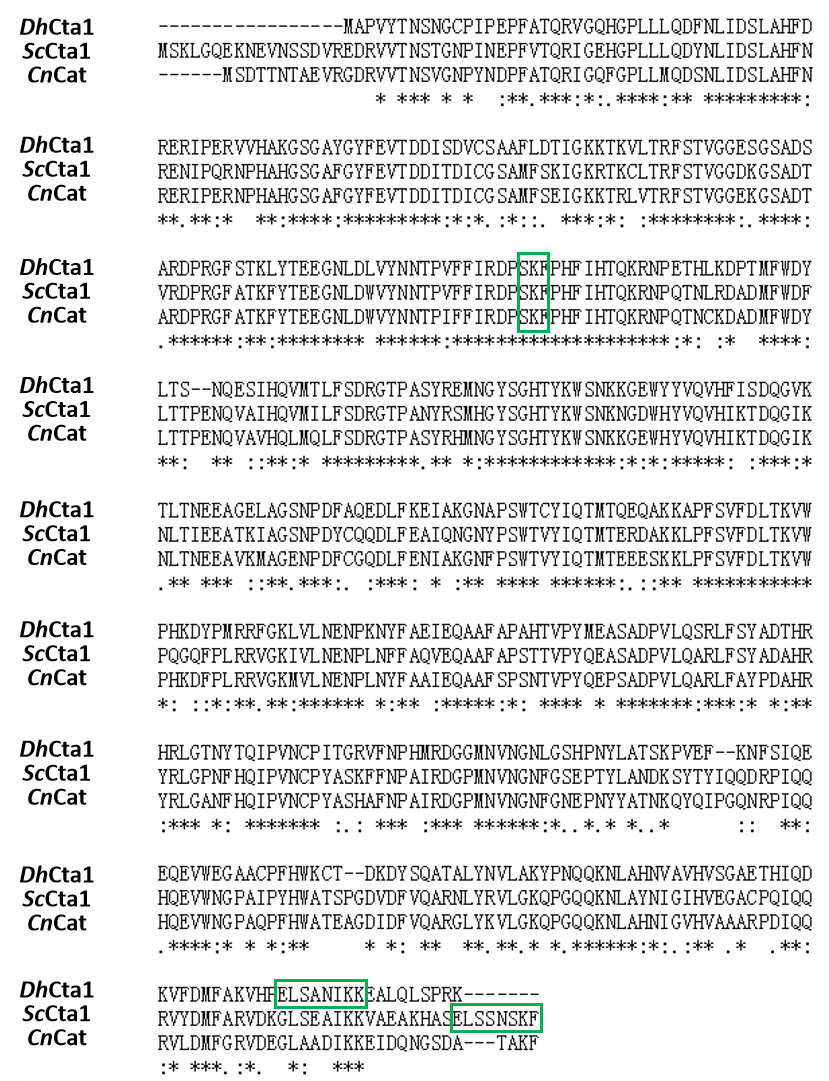


**Fig. S5 Comparative amino acid sequence analysis of catalase from *S. cerevisiae*, *D. hansenii* and *C. nivariensis*.** The asterisk (*) indicates identical amino acid sequences, while a colon (:) or a period (.) represent a strong or weak similarity, respectively. Speculated peroxisomal signal sequences are highlighted in green box.


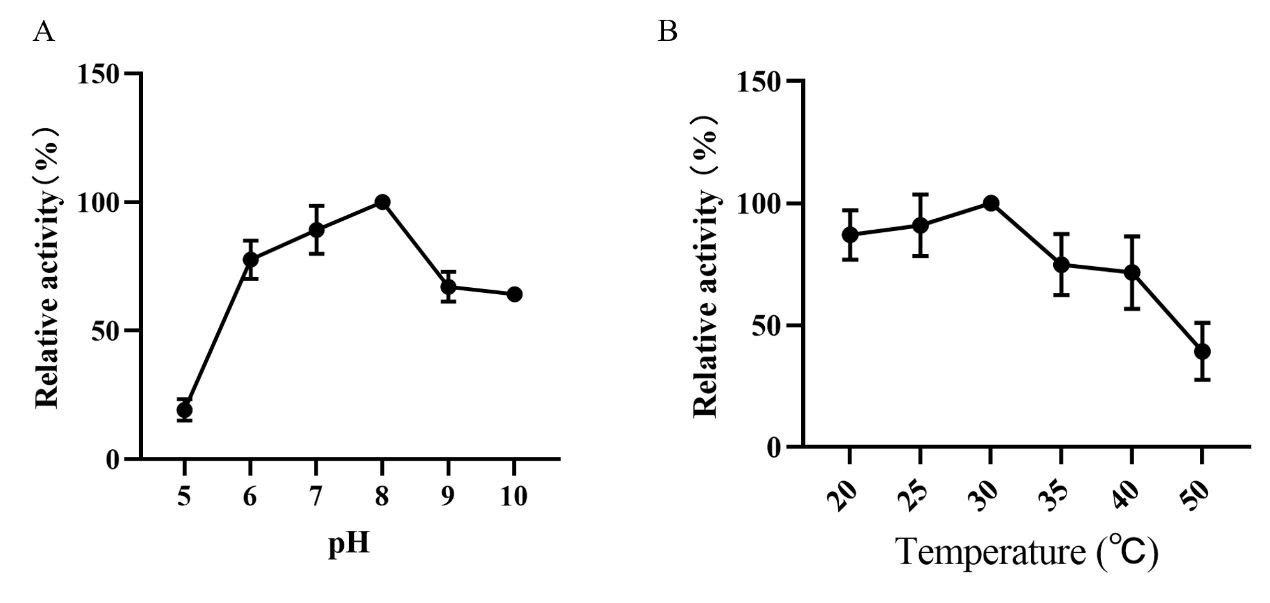


**Fig. S6 The** **optimal pH and temperature of His-*Cn*Cat.**

A Optimal pH of His-*Cn*Cat. The activities of the purified recombinant protein His-*Cn*Cat were measured in buffers with different pH values ranging from 5 to 10.

B Optimal temperature of His-*Cn*Cat. The activities of the purified recombinant protein His-*Cn*Cat were measured at different temperature ranging from 20 to 50 ℃.


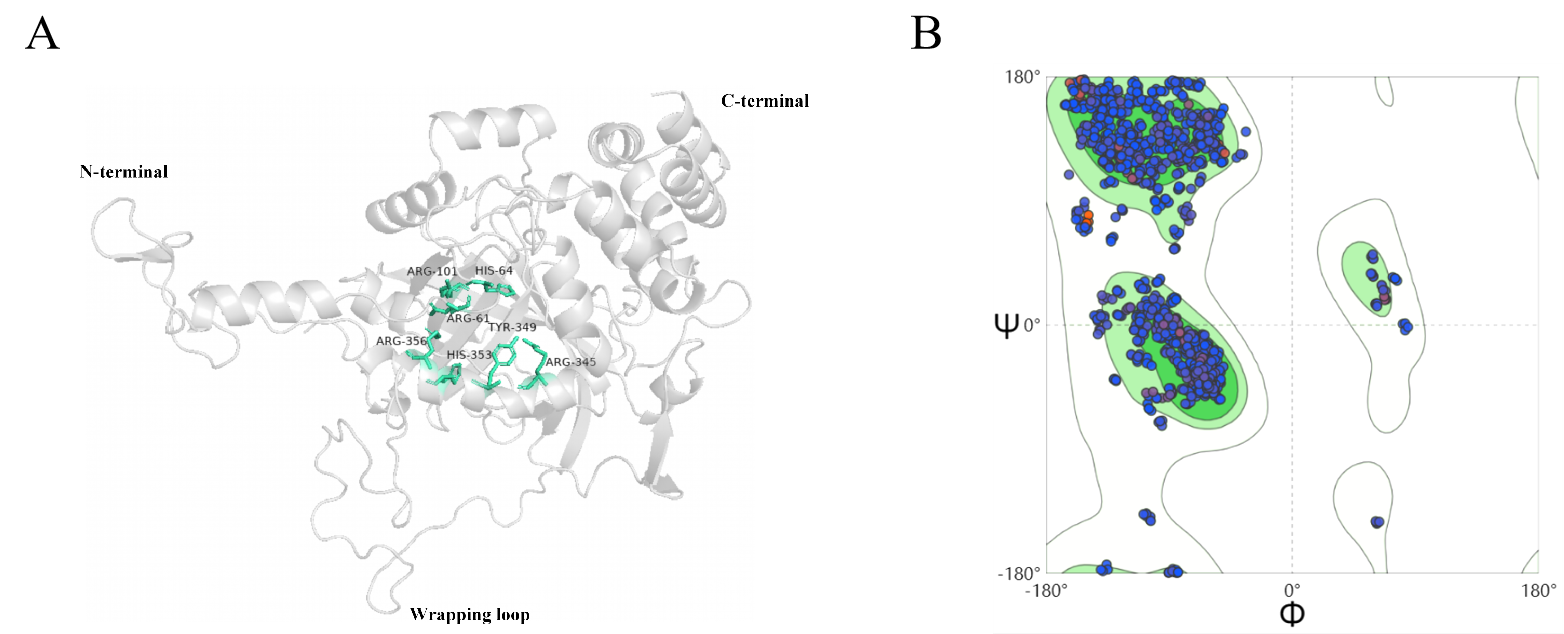


**Fig. S7 Homologous modeling of *Cn*Cat.**

A Protein structure of *Cn*Cat. The grey ribbon represents the monomer fold of catalase *Cn*Cat, and the green color indicates the active sites.

B Ramachandran plot of *Cn*Cat.

**Table S1** Primers used in this study.

| **Primers** | **Nucleotide sequence (5′→3′)** | **The amplified fragment or the utilization** |
| --- | --- | --- |
| *act1*-qRT-F/R | TGGATTCTGAAGTTGCTGC/GGGTAACGCAAGGTCAAGA | 210 bp DNA fragment of *act1*, used for qRT-PCR |
| *cag*-qRT-F/R | ATGGCTAGTGTTGCTGTTTC/AGTGCACAATACCAGCAGCA | 211 bp DNA fragment of *cag*, used for qRT-PCR |
| *cat*-qRT-F/R | ATGTCTGACACTACGAATACT/TGATATCATCGGTAACTTC | 242 bp DNA fragment of *cat*, used for qRT-PCR |
| *ccs1*-qRT-F/R | ATGACTGCTAATTCTGATTTCTACG/TTACCAGCACCTCTGATGAT | 227 bp DNA fragment of *ccs1*, used for qRT-PCR |
| *gcg1*-qRT-F/R | ATGACTGTTGCGAACGGTGG/CGTTGAGAAACCTGCGGTTA | 220 bp DNA fragment of *cag*, used for qRT-PCR |
| *hyr1*-qRF/R | ATGACTGCAGAGAGTGAATT/AAACTGATTACAGGGAAACC | 210 bp DNA fragment of *hyr1*, used for qRT-PCR |
| *trr1*-qRF/R | ATGAACCACAAGAGAGTTGT/AGAACCAGTCATACCATCTG | 195bp DNA fragment of *trr1*, used for qRT-PCR |
| *cag*-oeF/R | actatatcgtaataccatatgGCTAGTGTTGCTGTTTCTAGAAGTGTG/atggtgatgcacgtgcatatgCAAGTCTTGTTCGTCCAAGGTCT | 1110 bp DNA fragment of *cag*, used for construction of overexpression plasmid |
| *cat*-oeF/R | actatatcgtaataccatatgTCTGACACTACGAATACTGCTGAAGTT/atggtgatgcacgtgcatatgGAACTTGGCGGTGGCATCA | 1557 bp DNA fragment of *cat*, used for construction of overexpression plasmid |
| *ccs1*-oeF/R | actatatcgtaataccatatgACTGCTAATTCTGATTTCTACGAGGC/atggtgatgcacgtgcatatgTTTAATGTTGTTCTTTAGGGCGTC | 753 bp DNA fragment of *ccs1*, used for construction of overexpression plasmid |
| *gcg1*-oeF/R | actatatcgtaataccatatgACTGTTGCGAACGGTGGGA/atggtgatgcacgtgcatatgCTTGTTTTCTATATTCAGCGCGTT | 768 bp DNA fragment of *ccs1*, used for construction of overexpression plasmid |
| *hyr1*-oeF/R | actatatcgtaataccatatgACTGCAGAGAGTGAATTTTACAACCT/atggtgatgcacgtgcatatgTTTATTCTCTGAGTCTTGAAGGAGCT | 543 bp DNA fragment of *ccs1*, used for construction of overexpression plasmid |
| *trr1*-oeF/R | actatatcgtaataccatatgAACCACAAGAGAGTTGTTATTATCGG/atggtgatgcacgtgcatatgCTCCAATTCAGTCAAGTATCTTTCAGC | 996 bp DNA fragment of *ccs1*, used for construction of overexpression plasmid |
| P1/P2 | atcttccagagatAGGTGTCTCACGAACCATGGTAA/gggcctccatgtcTTACTAATGATCGGAATTGT | 963 bp DNA sequence upstream of *cat*, used for construction of *cat* deletion mutant |
| P5/P6 | ttagtaaGACATGGAGGCCCAGAATACC/caatCAGTATAGCGACCAGCATTCACA | 1120 bp DNA sequence of screening marker, used for construction of *cat* deletion mutant |
| P3/P4 | gctggtcgctatactgATTGTGTTGAATGAATTGTTC /tgccgttcgacgatGACAGCATCTGTAAAGAGAG | 962 bp DNA sequence downstream of *cat*, used for construction of *cat* deletion mutant |
| P7/P8 | TGGCGATACTGGCCAAGTCAGT/TCTTGAGCTCTAGCAAGTCTGGTTG | 3747 bp DNA sequence, used for PCR validation of *cat* deletion mutant |

**Table S2** Differential expressed under H_2_O_2_-stressed conditions.

| **ID** | **Gene** | **Log_2_FC** | | **Annotation** |
| --- | --- | --- | --- | --- |
|  |  | 20 vs 0 (mM) | 20 vs 2 (mM) |  |
| **Differential expressed antioxidant genes under H_2_O_2_-stressed conditions** | | | | |
| EVM0001060 | *cag* | 1.530798587 | n.s | Peroxidase |
| EVM0003487 | *gcg1* | 1.163234516 | n.s | Glutathione-specific gamma-glutamylcyclotransferase |
| EVM0001156 | *cat* | 2.1405951 | 1.078014875 | Peroxisomal catalase |
| EVM0000437 | *ccs1* | 1.558167470 | 1.080508242 | Copper/zinc superoxide dismutase |
| EVM0004844 | *hyr1* | 1.554615918 | 1.208068166 | Glutathione peroxidase |
| EVM0003781 | *trr1* | 5.221164267 | 1.268823894 | Pyridine nucleotide-disulphide oxidoreductase |
| **Differential expressed heat shock protein genes under H_2_O_2_-stressed conditions** | | | | |
| EVM0004531 | *sse1* | 1.107774069 | 1.113425002 | Heat shock protein homolog SSE1 |
| EVM0003579 | *ssa3* | 1.422918506 | 1.414982885 | Heat shock protein SSA3 |
| EVM0000829 | *hsc82* | 1.228004497 | 1.842565085 | ATP-dependent molecular chaperone HSC82 |
| EVM0004836 | *hsp60* | n.s | 1.051464437 | Heat shock protein 60 |
| EVM0004492 | *hsp20* | 1.068096637 | 1.41005258 | Endoplasmic reticulum chaperone BiP |
| EVM0003234 | *mreb* | 1.02514196 | 1.360040999 | Heat shock protein homolog SSE1 |
| EVM0002399 | *sse2* | n.s | 1.759205328 | Heat shock protein SSA2 |
| EVM0000117 | *hsp26* | 2.823559021 | 2.540040164 | Heat shock protein 26 |
| EVM0002922 | *hsp78* | n.s | 1.750222686 | Heat shock protein 78 |
| **Differential expressed ubiquitin pathway genes under H_2_O_2_-stressed conditions** | | | | |
| EVM0003580 | *uba1* | 1.318575536 | 1.307546825 | ubiquitin-activating enzyme E1 |
| EVM0003561 | *ubc8* | 1.468931518 | 1.399145489 | Ubiquitin-conjugating enzyme E2 |
| EVM0000483 | *ufd4* | 1.078539755 | 1.142299782 | Ubiquitin fusion degradation protein 4 |
| EVM0002550 | *ubc7* | 1.023002681 | n.s | Ubiquitin-conjugating enzyme E2 |
| EVM0002051 | *rad6* | 1.133985287 | n.s | Ubiquitin-conjugating enzyme E2 |
| **Differential expressed transcription factors under H_2_O_2_-stressed conditions** | | | | |
| EVM0004800 | *hac1* | 1.741334961 | 1.529284994 | Transcriptional activator HAC1 |
| EVM0003306 | *hta2* | -1.761203219 | -1.957536918 | Histone-like transcription factor |
| EVM0003039 | *mbf1* | 1.060721133 | 1.120090599 | Multiprotein-bridging factor 1 |
| EVM0002344 | *tec1* | 2.360314256 | 2.159104153 | Ty transcription activator TEC1 |
| EVM0002528 | *hcm1* | -1.333868498 | -1.502886811 | Forkhead transcription factor HCM1 |
| EVM0001621 | *pdr1* | 1.397742891 | 1.470778044 | Transcription factor PDR1 |

**Table S3** COG classification of regulated metabolic genes under H_2_O_2_-stressed conditions.

| Class_Name | 2 Vs 0 mM | 20 Vs 2 mM | 20 mM Vs 2 mM |
| --- | --- | --- | --- |
|  | Numbers | | |
| Translation, ribosomal structure and biogenesis | 1 | 86 | 68 |
| RNA processing and modification | 0 | 2 | 2 |
| Transcription | 0 | 8 | 9 |
| Replication, recombination and repair | 0 | 22 | 18 |
| Chromatin structure and dynamics | 0 | 1 | 0 |
| Cell cycle control, cell division, chromosome partitioning | 1 | 5 | 5 |
| Nuclear structure | 0 | 0 | 0 |
| Defense mechanisms | 1 | 10 | 7 |
| Signal transduction mechanisms | 2 | 22 | 17 |
| Cell wall/membrane/envelope biogenesis | 1 | 13 | 11 |
| Cell motility | 0 | 3 | 1 |
| Cytoskeleton | 0 | 1 | 1 |
| Extracellular structures | 0 | 0 | 0 |
| Intracellular trafficking, secretion, and vesicular transport | 0 | 1 | 1 |
| Posttranslational modification, protein turnover, chaperones | 5 | 84 | 74 |
| Energy production and conversion | 7 | 41 | 28 |
| Carbohydrate transport and metabolism | 7 | 67 | 56 |
| Amino acid transport and metabolism | 7 | 68 | 54 |
| Nucleotide transport and metabolism | 1 | 31 | 28 |
| Coenzyme transport and metabolism | 2 | 27 | 25 |
| Lipid transport and metabolism | 4 | 30 | 19 |
| Inorganic ion transport and metabolism | 5 | 30 | 23 |
| Secondary metabolites biosynthesis, transport and catabolism | 1 | 16 | 8 |
| General function prediction only | 6 | 64 | 53 |
| Function unknown | 0 | 11 | 8 |
| Mobilome: prophages, transposons | 0 | 0 | 0 |
